# Supplementary material for: Learning curve and surgical outcome of robotic assisted colorectal surgery with ERAS program
Source: Sci Rep. 2022 Nov 29;12:20566. doi: 10.1038/s41598-022-24665-w (PMC9709162; doi:10.1038/s41598-022-24665-w)
Supplement: Supplementary file 2 — Supplementary Information 2. [file 41598_2022_24665_MOESM2_ESM.zip › RAL Raw data-Ñ[▒K/080/HS_ñw╝╨░O▒Kñσ.pdf]

正式摘要(電子簽章：000000216715567)		
W55 / W55 ☐ 英文標題(注意:無英文姓名-請補病人基本檔!)	☐【讀取】憑證	

臺中榮民總醫院

出院病歷摘要

轉入號 : 轉出號 :

[redacted]

**(8)**流水編號	
**(9)**入院日期	110/05/05 (2021) 直腸外科 W55 - 282
**(10)**轉科日期	
**(11)**出院日期	110/05/12 (2021) 住院天數計 7 日

診斷

- 入院 : 1. Rectal cancer
  past hisotry:
  1. angina under medical tx
  2. GERD
  3. s/p appendectomy and CS
- 出院 : 1. Rectal cancer s/p robotic low anterior resection on 2021/05/06
  past hisotry:
  1. angina under medical tx
  2. GERD
  3. s/p appendectomy and CS

主訴

- colon cancer admitted for Robot Assisted Low Anterior Resection .

病史

- The 47-year-old female had underlying disease of
  1. angina under medical tx
  2. GERD
  3. s/p appendectomy and CS
  She underwent colonscope of health exam at CHOUTRAN Hospital and colon cancer was accidentally found and proved by pathology. She denied other symptoms except back soarness. Therefore, she was admitted for Robot Assisted Low Anterior Resection .

體檢發現

-
  - General appearance :
    - easy looking , conscious clear , without cardiopulmonary distress
    - Vital signs自動帶入date 體溫(BT) 脈搏(P) 呼吸(R) 血壓(BP) 身高(BH) 體重(BW) BMI
      20210505 168cm 73.2Kg 25.9
    - Vital signs: B.P. 136/94 mmHg , P.R. 108 /min , R.R. 18 /min , B.T. 37 ℃
  - HEENT :
    - Eyes: no anemic conjunctiva, no icteric sclera, normal EOM
    - ENT: no otorrhea, no rhinorrhea, no erythematous mucosa, no injected tonsil, no deformity
  - Neck: supple, freely movable, trachea midline,no deformity, no engorged jugular vein , no palpable thyroid or lymph node enlargement
  - Chest:
    - Inspection: Grossly normal, symmetric and fully expansion, no accessory muscle used, no operation scar, no spider angiomata, no gynecomastia
    - Palpation: no palpable mass or nodule, no subcutaneous emphysema, no tenderness
    - Ausculation: clear breathing sound
  - Heart:

- Percussion: normal heart size
  - Ausculation: regular heart beat, normal S1, S2, no gallop, no murmur
- Abdomen:
  - Inspection: in
    - flat contour, symetrically, no scar, no stria, no dilated veins, no rashes
  - Ausculation: normoactive bowel sound, no abdominal bruits
  - palpation: soft, no muscle guarding or rigidity, no local tenderness, no rebounding pain, no palpable mass, no palpable pulsation
  - Percussion: no shifting dulllness, no hepatomegaly, no splenomagaly
-

Peripheral pulsation :	CA	BA	RA	FA	PA	PTA	DPA
Right side :	++	++	++	++	++	++	++/+++
Left side :	++	++	++	++	++	++	++/+++

- Genito-anus: no tenderness or palpable mass lesion or rectal shelf, normal external genitalia, normal anal tone, brown stool

CRS CONDITION

-
  - Lung

手術日期及方法

- 手術日期及方法20210506 1. Robotic (XI) assisted low anterior resection
  2. Intraoperative colonoscopy : (+). The tumor position was checked again. It was already s/p tattoo for localization.
  3. High ligation (+) : At IMA root. Left colic artery and IMV were also ligated
  4. Take down from the splenic flexure : (-)
  5. Robotic endoGIA (45-4.1) X2 + EEA (33mm) were used
  6. Proximal cut end: > 10 cm, distal cut end: 1 cm
  7. No drainage tube was inserted
  8. ADHESION BARRIER FILM was placed below the fascia of the central umbilicus wound

住院治療經過

- After admission, robotic low anterior resection was done on 2021/05/06 under no contraindication. After operation, right lower limb numbness was complained and improved later on. Diarrhea was noted and improved after smecta use. Under stable condition, she was discharge and arranged OPD followed up.

合併症

一般檢查

- 累積報告 :

DATE	TIME	ALB	TP	BIL.T	BIL.D	ALKP	AST	ALT	LDH	CHOL	TG	HDL-C	LDL	UA	NH3	AMY
1100505	1909	4.7	7.4	1.0	0.2	99	32	48	172	180	241	44	94	5.3		
1100506	2326	3.7		1.1			32	44								
1100511	0851	3.9		0.9				38								

DATE	TIME	WBC	RBC	HGB	HCT	MCV	PLT	BLST	NEUT	BAND	SEG	LYM	MON	EOS	BAS
1100505	1909	6500	5.06	10.4	33.6	66.4	319		45.7			45.1	4.8	3.5	0.9
1100506	2016	13980		10.0	31.9		308		91.4			7.7	0.7	0.1	0.1
1100511	0859	7500	4.66	9.7			349		62.8			25.6	4.8	6.3	0.5

DATE	TIME	NA	K	CL	CA	BUN	CREAT	Mg	P	CRP	ESR	PCT	LIPASE	LACTATE
1100505	1909	141	3.5	104	9.1	15	0.64			0.055				
1100506	2326	141	3.8		8.7	10	0.52							
1100511	0851	142	3.8		8.9	8	0.56							

DATE	TIME	GLUCOSE	PAB	TRF	ZN	CU	KETONE	CK	CKMB	PT-P	PT-C	APTT-P	APTT-C	TRO-T
1100505	1909	79								10.0	10.6	25.4		

特殊檢查

病理報告

其它

出院時情況

- 3.改門診治療

出院指示

-

藥名	劑量	劑量單位	用法	頻次	數量	數量單位	現況	天數	單位
Xanax tab 0.5mg	0.5	TAB	PO	HS	4	TAB	TH	7	天
Herbesser tab 30mg	1	TAB	PO	BID	14	TAB	TH	7	天
Ultracet tab	1	TAB	PO	Q6HPRN	20	TAB	TH	5	天
$Biofermin tab	2	TAB	PO	TID	42	TAB	TH	7	天
Urea cream 10% 20g		TUB	TOP	QD	0	TUB	TH	1	瓶
(□)Takepron OD tab 30mg	1	TAB	PO	QDAC	7	TAB	TH	7	天
Strocain TAB	1	TAB	PO	BID	14	TAB	TH	7	天
Dobecon tab 100mg	1	TAB	PO	BIDAC	14	TAB	TH	7	天
Smecta powder 3g	1	PKG	PO	TIDPRN	21	PKG	TH	7	天

出院計劃

主治醫師_2062F 蔣鋒帆_______________ / 住院醫師_9760F 趙英如_______________

正式摘要**(**電子簽章：**000000216715567)**

W55 / W55

doctorRank:B
